# Influence of Active-to-Passive Ratio on the Deformation in Circular Dielectric Elastomer Actuators

**DOI:** 10.3390/mi15010125

**Published:** 2024-01-11

**Authors:** Markus Koenigsdorff, Hans Liebscher, Petr Osipov, Johannes Mersch, Gerald Gerlach

**Affiliations:** 1 Institute of Solid-State Electronics, Faculty of Electrical and Computer Engineering, TUD Dresden University of Technology, Mommsenstraße 15, 01069 Dresden, Germany; hans.liebscher@tu-dresden.de (H.L.); petr.osipov1@mailbox.tu-dresden.de (P.O.); johannes.mersch@tu-dresden.de (J.M.); gerald.gerlach@tu-dresden.de (G.G.); 2 Institute of Measurement Technology, Faculty of Mechatronics, Johannes Kepler University Linz, Altenberger Straße 69, 4040 Linz, Austria

**Keywords:** dielectric elastomer actuator, circular actuator, dot actuator, active-to-passive ratio, pre-stretch, hyperelastic model, lumped parameter model, electro-mechanical transducer

## Abstract

To further improve the performance of dielectric elastomer actuaotrs (DEAs), the development of novel elastomers with enhanced electro-mechanical properties is focal for the advancement of the technology. Hence, reliable techniques to assess their electro-mechanical performance are necessary. Characterization of the actuator materials is often achieved by fabricating circular DEAs with the pre-stretch of the membrane fixed by a stiff frame. Because of this set-up, the electrode size relative to the carrier frame’s dimension has an impact on actuator strain and displacement. To allow for comparable results across different studies, the influence of this effect needs to be quantified and taken into account. This paper presents an in-depth study of the active-to-passive ratio by proposing two simplified analytical models for circular DEA and comparing them. The first model is taking the hyperelastic material properties of the dielectric film into account while the second model is a linear elastic lumped parameter model based on the electro-mechanical analogy. Both models lie in good agreement and show a significant linear impact of the radial active-to-passive ratio on the electro-active strain and a resulting maximum of displacement around 50% radial coverage ratio. These findings are validated by experiments with actuators fabricated using silicone membranes. It is shown that the electrode size is not only an important parameter in the experimental design, but in some cases of higher significance for the accuracy of analytical models than the hyperelastic properties of the material. Furthermore, it could be shown that a radial coverage ratio of around 50% is desirable when measuring displacement as it maximizes the displacement and lowers the impact of deviations in electrode sizes due to fabrication errors.

## 1. Introduction

**Figure 1:**
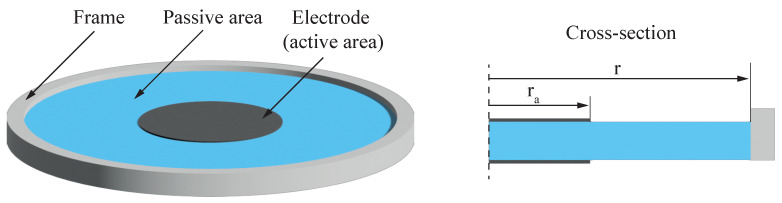
General structure of a circular DEA consisting of a pre-stretched dielectric elastomer fixed to a stiff frame with a circular hole. The centered electrodes are surrounded by an annular passive region.

In contemporary research and technological developments, dielectric elastomer actuators (DEAs) have gained considerable attention as a distinctive category of soft actuators. These DEAs, characterized by their capability to undergo substantial deformations in response to electrical stimuli, have positioned themselves as a compelling choice for a diverse array of engineering applications. Their unique combination of mechanical flexibility and electro-active properties distinguishes DEAs from traditional actuators, opening up new possibilities for the design and implementation of soft robotic systems. In a typical set-up, a DEA consists of a flexible capacitor structure. This structure has two soft electrodes separated by a stretchable dielectric elastomer (DE). When a voltage is applied across these electrodes, the DE compresses in the direction of the electric field, causing it to expand in two dimensions [1,2]. This expansion can be increased by initially pre-stretching the DE. To maintain the pre-stretch, various methods are used, such as adding weights [3], using pre-tensioned springs [4], or employing negative bias springs [5,6]. Another common approach is to attach the pre-stretched DE to a rigid frame as shown in Figure 1. This set-up allows the incorporation of a fixed DEA into mechanical systems and also allows comparing the electrical and mechanical properties of different DE materials [2,7,8]. Due to the ease of manufacturing such dot actuators and possibility to test them with a camera-based set-up, they have become the standard for evaluating novel dielectric and electrode materials both statically and dynamically [9]. Additionally, other influencing factors, e.g., humidity or pre-stretch are also regularly investigated with these set-ups [10,11]. However, when the stretched DE is fixed with a frame, instead of using biasing mechanisms, the electro-active strain of the DEA depends on the ratio between the active (with electrode coating) and passive (without electrode coating) areas of the actuator. Based on the schematic DEA cross-section in Figure 1, the radial active-to-passive ratio, also labeled as coverage ratio, *B*, is defined by

(1) $$B=\frac{r_{a}}{r},$$

where $r_{a}$ is the radius of the electrode and *r* the inner radius of the rigid frame that corresponds to the total DE radius.

Circular DEAs, or so-called dot actuators, with centered electrodes are widely used to assess the electro-mechanical performance of DEs or electrode materials by evaluating the electrode strain during actuation. Carpi et al. in their work “Standards for dielectric elastomer transducers” recommended to use actuators with electrode radii of one tenth to one third of the total DE radius [9]. However, this ratio specification might not be sufficient as a standard, since the wide range for electrode sizes means that actuation strain studies by different research groups can be difficult to compare. For example, different studies on the commercial, acrylic DE VHB 4910 show considerable differences in the ratio of the electrode radius to the inner radius of the carrier frame (total DE radius) as shown in Table 1 [12,13,14,15].

This is the first study which systematically examines the influence of the active-to-passive ratio of dot actuators both experimentally and theoretically. Therefore, dot actuators with varying pre-stretch and coverage ratios are prepared and electromechanically evaluated. Within this work, two simplified analytical models for the dot actuator are proposed that capture the influence of the electrode size on the measurable strain. The models are then compared to the experimental results. In general, the influence of the active-to-passive ratio of DEAs on their performance has major implications for the testing and evaluation of not only new electrode or DE materials but also for emerging applications. For example, tunable DEA-based lenses could be thought of as an inverse coverage ratio problem [16,17]. Therefore, given the feasibility of the presented models and experimental results, they could be used to predict and develop novel applications of DEAs as well.

## 2. Modeling

To analytically capture the electro-active response of DEAs, different models have been proposed. In the following, common modeling approaches are presented and compared to our proposed models to analyze the influence of the coverage ratio. For all presented models, the following general assumptions are taken:

- incompressibility of the dielectric material ( $\lambda_{1}\lambda_{2}\lambda_{3}=1$ ),
- independence of the relative permittivity $\varepsilon_{r}$ from material strain,
- negligible impact of electrodes on mechanical stiffness and
- homogeneity of the electric field *E*.

### 2.1. Pelrine Approach

**Figure 2:**
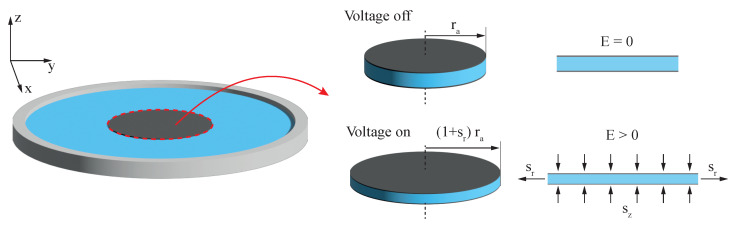
Schematic showing the Pelrine approach without consideration of pre-stretch resulting in a mechanical biasing load at electrode edges.

One of the commonly used approaches to calculate the deformation of DEAs is the Pelrine approach using linear elastic material properties [1,2,9]. As shown in Figure 2, it generally only accounts for the electrode area, neglecting forces stemming from the passive part, as illustrated in Figure 2.

The electro-active strain $s_{z}$ in thickness direction *z* is defined by

(2) $$s_{z}=-\frac{\varepsilon_{0}\varepsilon_{r}E^{2}}{Y},$$

where $\varepsilon_{0}$ is the permittivity in vacuum, $\varepsilon_{r}$ is the relative dielectric constant of the elastomer, *E* is the nominal electric field strength, and *Y* is the Young’s modulus of the elastomer. The calculated strain in thickness direction $s_{z}$ can be related to radial strain $s_{r}$ through the incompressibility of the elastomer. As under equi-biaxial conditions $s_{x}\;=\;s_{y}\;=\;s_{r}$ must hold, it follows that

(3) $$1=\left(1+s_{x}\right)\left(1+s_{y}\right)\left(1+s_{z}\right)={\left(1+s_{r}\right)}^{2}\left(1+s_{z}\right).$$

Hence, radial strain $s_{r}$ can be calculated by

(4) $$s_{r}=\frac{1}{\sqrt{1+s_{z}}}-1=\frac{1}{\sqrt{1-\frac{\varepsilon_{0}\varepsilon_{r}E^{2}}{Y}}}-1.$$

Because only the active part is considered, the electrode size has no influence on the calculated strain. To account for the effect of pre-stretching, the Young’s modulus is chosen at the operating point. Furthermore, the elastomer thickness is considered in the calculation of the electric field strength, which is expressed by applied voltage divided by elastomer layer thickness.

### 2.2. Hyperelastic Membrane under Constant Equi-Biaxial Force

To model large deformations, the hyperelastic properties of the material should be taken into account. The equations of state for an incompressible, ideal dielectric elastomer as proposed by Suo et al. are as follows [18]:

(5) $$\sigma_{1}-\sigma_{3}=\lambda_{1}\frac{\partial W_{stretch}\left(\lambda_{1},\lambda_{2}\right)}{\partial \lambda_{1}}-\varepsilon E^{2},$$

(6) $$\sigma_{2}-\sigma_{3}=\lambda_{2}\frac{\partial W_{stretch}\left(\lambda_{1},\lambda_{2}\right)}{\partial \lambda_{2}}-\varepsilon E^{2}.$$

In these equations, $W_{stretch}\left(\lambda_{1},\lambda_{2}\right)$ constitutes the Helmholtz free energy associated with the stretching of the elastomer. In the case of an equi-biaxial stretch state, $\lambda_{1}=\lambda_{2}=\lambda$ holds. Consequently, the 3-direction is chosen to coincide with the direction of the electric field. Therefore, it is assumed that $\sigma_{3}=0$ , because the surface of the DEA is unconstrained. Equations (5) and (6) of state simplify to ( (1,2,3)→(r,θ,z) ):

(7) $$\sigma_{r}=\lambda \frac{\partial W_{stretch}\left(\lambda_{r}=\lambda ,\lambda_{\theta }=\lambda \right)}{\partial \lambda_{r}}-\varepsilon E^{2}.$$

**Figure 3:**
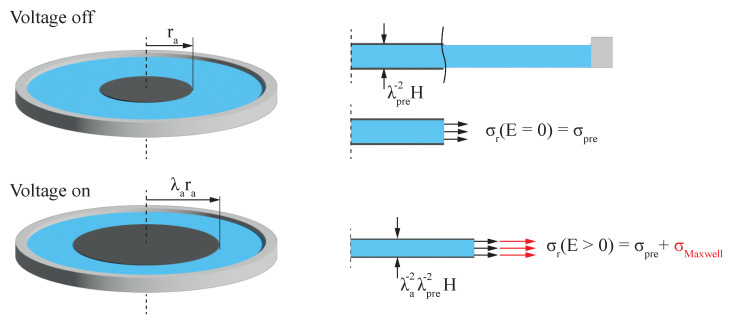
Schematic of the hyperelastic material model with constant mechanical load at the electrode edges due to strain independence of mechanical biasing load $\sigma_{r}$ .

As depicted in Figure 3, $\sigma_{r}$ accounts for constant external loads on the edge of the dielectric. As stated in Carpis standards for DEAs [9], to characterize dielectric films dot actuators with small electrode sizes with radii of 1:10 to 3:10 of the inner frame radius should lead to a behavior similar to that of a constant force acting on the edges of the electrode.

Introducing a constant biasing force *P* into the equation of state gives

(8) $$\sigma_{r}=\lambda \frac{P}{H2\pi R}=\lambda \frac{\partial W_{stretch}\left(\lambda_{r}=\lambda ,\lambda_{\theta }=\lambda \right)}{\partial \lambda_{r}}-\varepsilon E^{2}.$$

Here, *R* is the inital radius of the electrode and *H* is the initial thickness of the DE before pre-stretch and application of voltage. To reduce the complexity for the following calculations, the constant force is expressed as an equivalent constant pre-stretch in the passive area. Without an applied electric field, stretch ratio λ must be equivalent to applied pre-stretch $\lambda_{pre}$ . Therefore, setting E=0 in Equation (8) leads to

(9) $$\sigma_{r}\left(E=0\right)=\lambda_{pre}\frac{P}{H2\pi R}=\lambda_{pre}\frac{\partial W_{stretch}\left(\lambda_{r}=\lambda_{pre},\lambda_{\theta }=\lambda_{pre}\right)}{\partial \lambda_{r}}.$$

Hence, the equation of state for an ideal dielectric DEA under equi-biaxial constant loading follows by combining Eqaution (7) with (9):

(10) $$0=\lambda \frac{\partial W_{stretch}\left(\lambda_{r}=\lambda ,\lambda_{\theta }=\lambda \right)}{\partial \lambda_{r}}-\varepsilon E^{2}-\lambda_{pre}\frac{\partial W_{stretch}\left(\lambda_{r}=\lambda_{pre},\lambda_{\theta }=\lambda_{pre}\right)}{\partial \lambda_{r}}.$$

Within this contribution, the 3-parameter Yeoh material model is used for the description of DE properties [19]:

(11) $$\begin{array}{cc} W(I_{1}) & =\sum_{i=1}^{3}C_{i}{\left(I_{1}-3\right)}^{i}. \end{array}$$

For incompressible materials, this model is a function of the first strain invariant $I_{1}=\lambda_{1}^{2}+\lambda_{2}^{2}+\lambda_{3}^{2}$ and the three material parameters $C_{1}$ , $C_{2}$ and $C_{3}$ that capture the hyperelastic properties of the DE material. For the equi-biaxial stretched dot actuator, the stretch function follows

(12) $$\begin{array}{cc} W_{stretch}\left(\lambda_{r},\lambda_{\theta }\right) & =\sum_{i=1}^{3}C_{i}{\left(\lambda_{r}^{2}+\lambda_{\theta }^{2}+\lambda_{r}^{-2}\lambda_{\theta }^{-2}-3\right)}^{i}. \end{array}$$

Performing the differentiation in Equation (10) and setting $\lambda_{r}=\lambda_{\theta }=\lambda$ leads to the final equation of the model:

(13) $$\begin{array}{c} 0=2\left(\lambda^{2}-\lambda^{-4}\right)\left(C_{1}+2C_{2}\left(2\lambda^{2}+\lambda^{-4}-3\right)+3C_{3}{\left(2\lambda^{2}+\lambda^{-4}-3\right)}^{2}\right)-\varepsilon_{0}\varepsilon_{r}\lambda^{4}\frac{U^{2}}{H^{2}}\\ -2\left(\lambda_{pre}^{2}-\lambda_{pre}^{-4}\right)\left(C_{1}+2C_{2}\left(2\lambda_{pre}^{2}+\lambda_{pre}^{-4}-3\right)+3C_{3}{\left(2\lambda_{pre}^{2}+\lambda_{pre}^{-4}-3\right)}^{2}\right). \end{array}$$

The model with constant pre-stretch gives an upper bound for the active strain and displacement, as the constant force should coincide with the assumption of a negligible decrease in force due to released pre-stretch in the passive area. Furthermore, if $\lambda_{pre}=1$ is set, the mechanical biasing term vanishes because of term $(\lambda_{pre}^{2}-\lambda_{pre}^{-4})=0$ .

### 2.3. Hypereleastic Membrane under Equi-Biaxial Forces Considering Coverage Ratio

The model described in Section 2.2 considers mechanical biasing stress $\sigma_{r}$ as a constant value independent of the electro-active stress state. This simplification does not hold for large deformations or coverage ratios, as it is to be expected that the stress in the passive region drops significantly. It is possible to analytically fully describe the stress state in the passive region, but due to the non-uniformity of the stress state, the needed calculations are intensive [20]. So the approach presented here assumes an equi-biaxial stress state in the passive region. This is validated against experimental data.

Because the stiffness of the electrodes is not considered in the model, one can assume the same stiffness of the dielectric over the whole clamped radius. By defining the stretch ratio in active and passive area separately, the equation of the ideal DE can be formulated as

(14) $$0=\lambda_{A}\frac{\partial W(\lambda_{r}=\lambda_{A},\lambda_{\theta }=\lambda_{A})}{\partial \lambda_{r}}-\varepsilon E^{2}-\sigma_{r}\left(\lambda_{P}\right).$$

**Figure 4:**
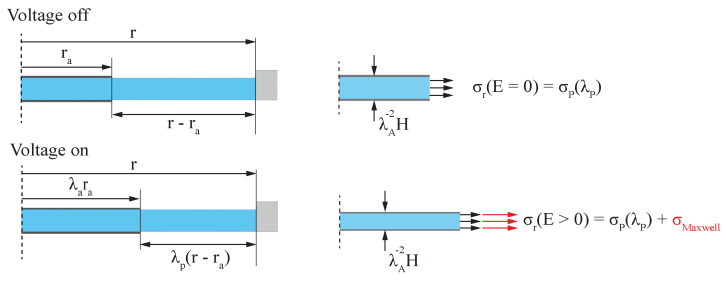
Schematic showing the hyperelastic model of the circular DEA with consideration of the coverage ratio *B* using the strain dependence of biasing stress $\sigma_{r}$ .

Here, $\lambda_{A}=\lambda_{a}\lambda_{pre}$ is the stretch in the active region and $\lambda_{P}=\lambda_{p}\lambda_{pre}$ is the resulting stretch in the passive part of the actuator. Mechanical stress $\sigma_{P}$ is acting at the electrode edge and is, in contrast to the previous model presented in Section 2.2, dependent on electrode deformation. The results of the boundary condition of the dot actuator are illustrated in Figure 4. Due to the simplification that the electrode does not influence the material stiffness, it is possible to express $\sigma_{P}$ through

(15) $$\sigma_{P}=\lambda_{P}\frac{\partial W(\lambda_{r}=\lambda_{P},\lambda_{\theta }=\lambda_{P})}{\partial \lambda_{r}}.$$

Expression $\lambda_{P}$ is the stretch ratio in the passive area that decreases with increasing strain in the active area, because the sum of electrode radius $r_{a}$ and the width of the passive material must equal to frame radius *r*. Figure 4 shows the geometric relation between the active and the passive region. Because the edge of the passive region is fixed geometrically, the following equation must hold:

(16) $$1=B\lambda_{a}+\left(1-B\right)\lambda_{p},$$

yielding

(17) $$\lambda_{P}=\lambda_{pre}\frac{1-B\lambda_{a}}{1-B}.$$

Now, it is possible to express the stretch in the passive area through the stretch in the active area leading to the final equation:

(18) $$\begin{array}{c} 0=2\left(\lambda_{A}^{2}-\lambda_{A}^{-4}\right)\left(C_{1}+2C_{2}\left(2\lambda_{A}^{2}+\lambda_{A}^{-4}-3\right)+3C_{3}{\left(2\lambda_{A}^{2}+\lambda_{A}^{-4}-3\right)}^{2}\right)-\varepsilon_{0}\varepsilon_{r}\lambda_{A}^{4}\frac{U^{2}}{H^{2}}\\ -2\left(\lambda_{P}^{2}-\lambda_{P}^{-4}\right)\left(C_{1}+2C_{2}\left(2\lambda_{P}^{2}+\lambda_{P}^{-4}-3\right)+3C_{3}{\left(2\lambda_{P}^{2}+\lambda_{P}^{-4}-3\right)}^{2}\right). \end{array}$$

When setting B=0 , the contribution of the passive area is independent of active stretch $\lambda_{a}$ because $\lambda_{p}=1$ . Thus, it follows that $\lambda_{P}=\lambda_{pre}$ , and the model transitions into the model with constant pre-stretch.

### 2.4. Linear Elastic Lumped Parameter Model

**Figure 5:**
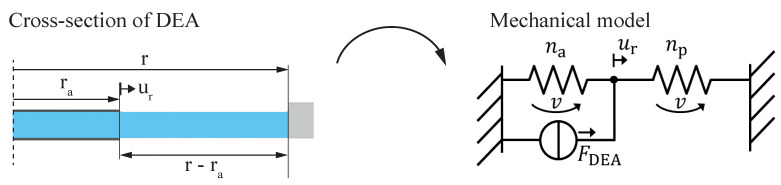
Derivation of the linear elastic mechanical network model for circular DEA behavior.

The lumped parameter model is derived by modeling a slice of the circular actuator with a network based on a mechanical structure for one-dimensional DEAs with a uniaxial strain state [21,22]. As shown in Figure 5, the active and the passive areas are described by their respective spring compliances $n_{a}$ and $n_{p}$ with velocity *v* and radial displacement $u_{r}$ .

Within this, the active area corresponds to a tension spring and the passive area to a compression spring. The respective compliances are given by

(19) $$n_{a}=\frac{r_{a}}{YA_{cross}}$$

and

(20) $$n_{p}=\frac{r-r_{a}}{YA_{cross}},$$

where *Y* is the Young’s modulus of the equi-biaxially stretched dielectric at the respective operating point and $A_{cross}$ is the stretch state-dependent cross-sectional area of the slice. The DEA force is represented by a load source having a value defined by

(21) $$F_{DEA}=\sigma_{Maxwell}A_{cross}=\varepsilon_{0}\varepsilon_{r}\lambda_{pre}^{4}\frac{U^{2}}{H^{2}}A_{cross}.$$

From the network shown in Figure 5, the following analytical solution can be obtained for electro-active strain $s_{a}$ :

(22) $$s_{a}=\left(1-B\right)\varepsilon_{0}\varepsilon_{r}\lambda_{pre}^{4}\frac{U^{2}}{H^{2}}\frac{1}{Y}.$$

In this, *Y* is set to be the Young’s modulus for the equi-biaxial stretch state at operating point $\lambda_{pre}$ . Because $r_{a}=Br$ , displacement $u_{r}$ can be calculated by

(23) $$u_{r}=B\left(1-B\right)\varepsilon_{0}\varepsilon_{r}\lambda_{pre}^{4}\frac{U^{2}}{H^{2}}\frac{r}{Y}.$$

This equation clearly shows a quadratic dependence of the electrode edge displacement on the coverage ratio, indicating a maximum in displacement at a radial coverage ratio of 50%.

## 3. Experimental Validation

### 3.1. Manufacturing of the Actuators

**Figure 6:**
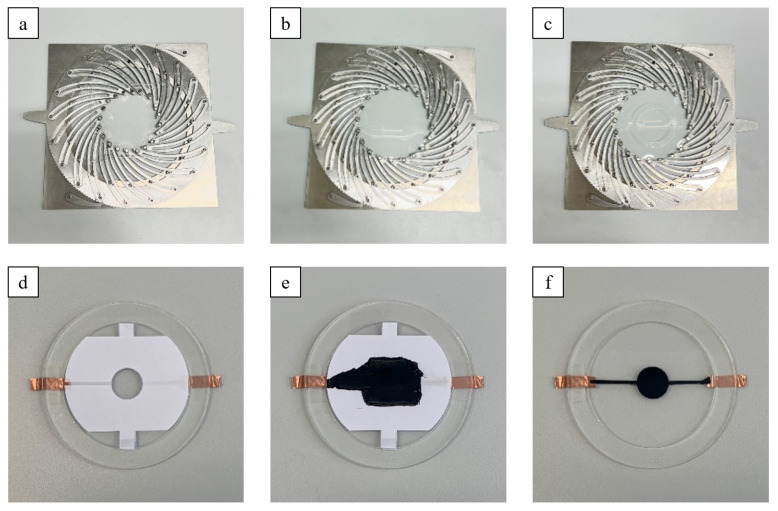
Manufacturing steps of the actuators: (**a**) Fixation of the dielectric film to the pre-stretcher with magnets, (**b**) pre-stretching of the film, (**c**) bonding of the carrier frame with double-sided adhesive tape to fix pre-stretch, (**d**) mask for electrode application, (**e**) carbon grease applied to dielectric film and (**f**) finished actuator.

In order to validate the presented models, circular DEAs were manufactured from 100 µm thick silicone film ELASTOSIL^®^ Film 2030 250/100 (Wacker Chemie AG, Munich, Germany). The Elastosil film was equi-biaxially pre-streched by ratios of $\lambda_{pre}$ = 1.1, 1.2 and 1.3, respectively, using a self-built pre-stretching device as shown in Figure 6. A circular acrylic frame with a inner diameter of 6 cm was bonded with a double-sided adhesive tape Adhesive Research^®^ EL-8932EE (Adhesive Research Inc., Glen Rock, PA, USA) on the pre-stretched silicone films. After bonding, the film material outside the frame was cut off. Subsequently, circular electrodes made of carbon grease NYOGEL 756G (Nye Lubricants Inc., Fairhaven, MA, USA) were applied with masks and spatula to both sides of the clamped silicone films. The electrode application and the one finished actuator are shown in Figure 6.

To study the influence of the active-to-passive ratio, the size of the circular electrodes has to be varied. For this purpose, masks with different circle cut-outs were used. For each DEA, the radial active-to-passive ratio was varied in the range from 10% to 90% in 5% increments. The respective pre-stretch was kept constant during the electrode deposition over the whole ratio range. The circular electrodes of the DEAs have connecting strips made of respective carbon grease, which lead to a copper adhesive tape that is attached to the acrylic frame. This copper tape serves as the terminal for high-voltage power supply.

### 3.2. Electro-Mechanical Characterization

**Figure 7:**
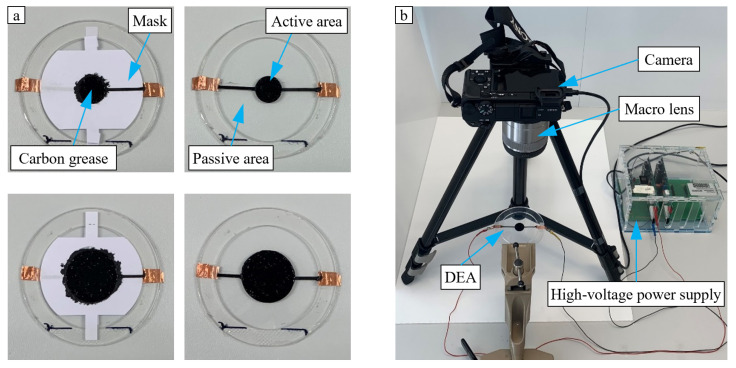
(**a**) Ratios 25% (upper) and 50% (lower) and (**b**) measurement set-up for DEA strain.

The influence of the active-to-passive ratio was investigated at three differently pre-stretched DEAs of Elastosil. Radial ratios of 25% and 50% applied on an Elastosil-based DEA are illustrated in Figure 7a as examples of coverage ratio variation during electro-mechanical characterization. Figure 7b shows the experimental set-up used to operate a DEA and record its electrically induced electrode deformation. The DEAs were driven with a constant voltage level of 4500 V using a Peta-pico-Voltron power supply [23]. In order to evaluate the electrode strain as a function of the coverage ratio, at each ratio, a 25 s video was recorded while the voltage was switched on and off. A compact camera (Sony α6400, Sony, Tokyo, Japan) with a macro lens (Sony SEL30M35, Sony, Tokyo, Japan) was used for video recordings at a speed of 100 frames per second. The distance between the lens and the observed electrode amounted to 12.5 cm. During the first 5 s of the video recording, the DEA was in a zero-voltage state to capture the initial electrode area. Constant voltage was then applied for 10 s, which lead to an equi-biaxial expansion of the electrode area, corresponding to a radial electrode strain. Subsequently, the voltage was switched off and the video recording continued for 10 s to track the recovery behavior of the electrode region.

MATLAB (Version 9.10 (R2021a), The MathWorks Inc., Natick, MA, USA) was used for video processing and the pixel-based extraction of the time-discrete area strain, from which the radial strain and displacement were calculated. For this, the electrode area was considered as an ideal circular surface. To minimize the influence of the contacting strips, the frames were cut to the relevant region of interest showing only the electrode. In order to determine one strain value $s_{r}$ and one displacement value $u_{r}$ for each coverage ratio, the time-discrete strain and displacement signals were averaged in the “voltage on” state between 6 s and 8 s.

## 4. Results

### 4.1. Model Results

The mechanical properties of the dielectric silicone membrane are described by a set of three parameters which are calculated by fitting the hyperelastic Yeoh material model onto equi-biaxial and uniaxial data supplied by the manufacturer of the film. Fitting the parameters onto multiple stretch states improves the overall accuracy of the hyperelastic model [24,25]. Table 2 lists the used parameters.

**Figure 8:**
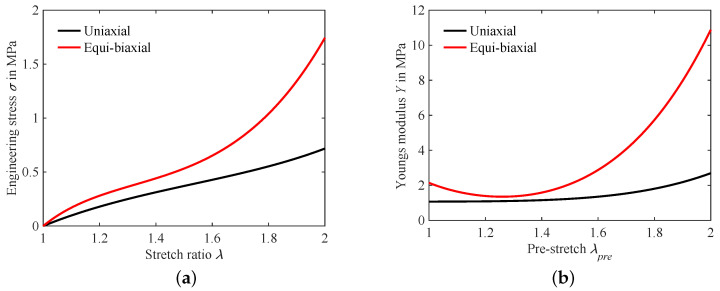
Hyperelastic behavior of DE material: (**a**) Stretch strain curves for uniaxial and equi-biaxial case and (**b**) influence of pre-stretch on Young’s modulus.

The engineering stress for the stretch ratios up to λ=2 are shown in Figure 8a. As expected, the stress for the equi-biaxial stretch state is significantly higher then in the uniaxial stretch-state. Furthermore, the membrane shows clear stiffening behavior setting in around λ=1.6 . This becomes more evident when looking at the progression of Young’s modulus *Y* over the stretch state, which is shown in Figure 8b. Additionally, this plot illustrates the initial softening of the material, which is a driving factor in the influence of pre-stretch on the improvement of electro-active strain in DEAs. The values shown in this plot are then used as the stiffness in the linear models presented in Section 2.1 and Section 2.4 for the circular DEA.

**Figure 9:**
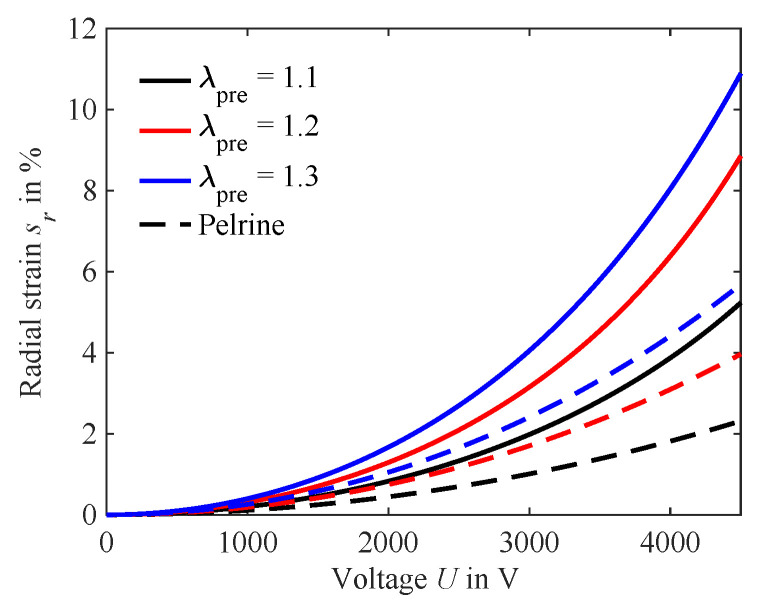
Electro-active strain calculated with hyperelastic model under constant force (Equation (13)) and Pelrine approach (Equation (4)).

As described in Section 2.1, a typical approach is to calculate the radial strain of a circular DEA with Equation (4). The results of this calculation are compared against the hyperelastic model with constant force from Section 2.2 in Figure 9.

For each level of pre-stretch, Young’s modulus for the equi-biaxial operating point is calculated and used with Equation (4). For the calculation of Maxwell pressure, $\varepsilon_{r}=2.8$ , as specified by the manufacturer, is used. Qualitatively, both approaches show a similar response to the voltage, with a quadratic increase in the strain with voltage. However, as to be expected, implementing a constant pre-stretch as a radial biasing force significantly increases the calculated strain. This is the equivalent to the following results for virtual coverage ratio B=0 .

**Figure 10:**
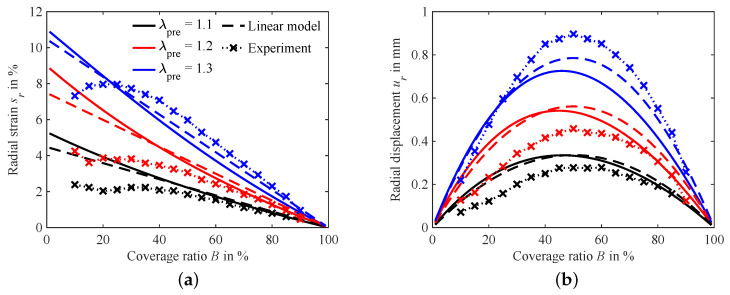
Comparison of the experimental results with the hyperelastic and lumped parameter modeling results of Section 2.3 and Section 2.4: (**a**) on strain and (**b**) on displacement.

Using the hyperelastic coverage ratio model from Section 2.3 and the lumped parameter model from Section 2.4, the plots shown in Figure 10 are calculated. Both models show a decrease in the electrode strain with rising coverage ratio. As expected, *B* has a linear influence on the strain in the lumped parameter model, but the hyperelastic model does not deviate significantly from the linear solution. For coverage ratios below 20%, the linear model is consistently below the results of the hyperelastic model. However, this effect decreases for higher levels of pre-stretch. The reason can be found when performing comparison to the aforementioned mechanical properties of the membrane illustrated in Figure 8b. Around the highest investigated level of pre-stretch of $\lambda_{pre}=1.3$ , Young’s modulus is nearly constant, hence the material properties resemble those of a linear elastic material.

The calculated displacements depicted in Figure 10b also show only minor deviations, as expected due to the similar strain results of the two models. For the linear elastic lumped parameter model, the maximal displacement always lies at a coverage ratio B=50% , as is clearly deducible from Equation (23). In contrast, the maximum displacement in the hyperelastic model is shifted slightly towards coverage ratios lower than 50% for all modeled pre-stretch levels.

### 4.2. Experimental Results

Figure 10a compares the measured electro-active strain of actuators with three different levels of pre-stretch to the model results. Strikingly, the model results are in good agreement with the measurements, except for lower levels of coverage, where they significantly deviate. For all measured samples, the maximal strain in these areas is significantly lower than expected, and it does not follow the expected nearly linear trend. A possible explanation for this can be found in manufacturing errors. For small electrode sizes, the correct alignment of both electrodes is more difficult, and small alignment errors have a relatively higher impact on the active area and therefore the strain. Additionally, as the camera is not moved closer to the sample between the measurements and no optical zoom is used, the measurement resolution for smaller electrodes in turn is also lesser. This results in a higher signal-to-noise ratio for these measurements, which is detrimental to the accuracy of the measurement. Nonetheless, the influence of the coverage ratio on the achievable strain is clearly visible, hence verifying the importance of considering this parameter in experimental designs and models.

The displacement calculated from these measurements shown in Figure 10b validates the expected peak of displacement around B=50% . Due to deviations in strain measurements, the displacement curves are not symmetrical, as lower strains for small coverages lead to a flattening of the left side of the curve. With the models primarily deviating from each other in that area, it is not possible to come to a conclusive decision as to which model more closely fits the experimental results. It is notable that for all pre-stretch levels except for $\lambda_{pre}=1.3$ , the measured displacements are lower than the model results. This is the intuitive result, as the influence of the electrode paste is not considered in the model. However, for the pre-stretch of $\lambda_{pre}=1.3$ , the maximum of the data lies in nearly perfect agreement with the linear model results and slightly above the predicted maximal displacement of the hyperelastic model. A possible explanation for this is that small variations in the Yeoh parameters used to predict the stresses inside the material are over-proportionally amplified by stretch. Hence, for higher stretches, the deviations between model and experiment might be increased.

## 5. Discussion

Both the hyperelastic model (Equation (18)) and the linear lumped parameter model (Equation (22)) show a significant influence of the electrode size and the resulting coverage ratio on the elctro-active strain and displacement. These findings are experimentally validated by the fabrication of dot actuators from equi-biaxially pre-stretched Elastosil films. Using carbon grease as electrodes, different coverage ratios can be achieved. The mechanical properties of the DE material are captured by the hyperelastic Yeoh model.

In both models, the electro-active strain decreases with the raising coverage ratio. This is an intuitive result, as for a coverage ratio approaching 0% the electro-active strain has to be maximal. This is due to the biasing force provided by the pre-stretch in the passive area around the electrode not being able to decrease in this case, as no pre-stretch can be released. This is also analytically described with the model presented in Section 2.2. On the other hand, the electro-active strain must be zero in case of a coverage ratio of 100% as the active areas edges are clamped and therefore prevented from deforming. Both models show these phenomena; however, the experimental strain data deviate significantly from the model predictions for smaller coverage ratios.

As already touched on in Section 4.2, there are several potential reasons that could explain these deviations. For once, any alignment errors that arise in the fabrication of the actuator become more significant for smaller electrode sizes. However, the consistent presence of these deviations across all measured actuators hints at a systematic error in the experimental set-up. As described in Section 3.2, the strain of the dot actuators is extracted by using a MATLAB script tracking the increase in black pixels in each video frame when the electrode deforms. The area increase in the black pixel region is then used to calculate an equivalent radial strain assuming a circular electrode. As visible in Figure 7, the circular electrodes are contacted with a thin strip of carbon grease. During the analysis, the frames are cut to the relevant region of interest showing only the electrode, but as the width of the strip stays constant through all measurements, errors introduced due to it influence smaller electrode radii stronger. Furthermore, the experiments are all recorded with the same level of optical zoom, which results in a worse resolution for smaller electrodes, further impacting these measurement results. In future experiments, alternative approaches should be considered; for example, measuring the electrode edge displacement with a laser sensor.

Nonetheless, it is clearly visible that both models are able to predict the strain and displacement, also when considering varying levels of pre-stretch. Interestingly, the hyperelastic model (Equation (18)) and the linear elastic model (Equation (22)) do not deviate significantly for the DE material used within this study. Especially compared to the differences to the conventional linear elastic and hyperelastic approaches presented in Section 2.1 and Section 2.2, the importance of the coverage ratio as a simulation parameter becomes evident. Even though a generalization for all DE materials based solely on this study is not accurate, within the scope of this work, the consideration of the coverage ratio is of higher significance than that of hyperelastic material properties. Therefore, the linear elastic lumped parameter model (Equation (22)) provides a powerful and simple-to-implement analytical approach to model the electro-active strain in dot actuators.

The results from both experimental investigations and simulations reveal that the maximal displacement occurs when the active-to-passive ratio is set at 50%, even when varying the pre-stretch level. In this ratio range, the parabolic displacement curves exhibit minimal slopes compared to lower or higher coverage ratios. Consequently, the sensitivity to manufacturing irregularities in dielectric elastomer actuator (DEA) production and uncertainties in electro-active strain measurements is at its lowest within this range.

For example, an unintentionally smaller electrode with a coverage ratio of 48% instead of the intended 50% leads to a reduction in displacement by 0.16%. In comparison, a proportional error in the coverage ratio of 24% instead of 25% results in an error of 2.72%.

It is noteworthy that the active-to-passive ratio range of 10% to 30%, as suggested by Carpi et al. [9] for the circular DEAs studied here, results in non-constant actuation strain and displacement. This emphasizes that performance studies conducted with different active-to-passive ratios are not comparable, even when the DE pre-stretch and film thickness are uniform. Consequently, based on the coverage ratio alone, the measured strain of VHB varies by a factor of >3 between the studies in Table 1. Although alternative measurement methodologies such as laser triangulation scanners can increase the accuracy in comparison with the camera-based approach used in this study, the error introduced by varying electrode size and the resulting limited comparability persist.

Based on experimental and model results, we propose that for the characterization of DE materials with dot actuators a radial coverage ratio of about 50% should be aimed to maximize displacement. Furthermore, this ratio will decrease the influence of electrode size, for example, by variations in fabrication, on the measured displacement. However, in the case of DE materials with high electro-active strains, the electrode size must possibly be reduced to prevent instabilities due to the loss of tension in the passive area during activation. In any case, as the electro-active strain reduces to 0% at 100% coverage, we could calculate the theoretical strain at 0% coverage using a linear fit with an arbitrary electrode size. This would allow the comparison of experimental results of actuators fabricated with varying coverage ratios. Variations in pre-stretch, thickness and modulus are parameters of the model and would need to be chosen to be equal. Alternatively, two set-ups can be compared by using the presented models.

In addition to the implication this study has for dielectric elastomer testing and characterization, the developed models could also be used to simulate and design DEA-based tunable lenses [26]. These often consist of an inverse dot actuator with the electrode covering the space between frame and transparent lens in the center. The transparent lens can be seen as the passive area [27]. By expanding the equivalent circuit model to include the visco-elastic behavior of the elastomers, our approach can model not only the static but also the dynamic behavior of such ultrafast lenses [17,28].

## 6. Conclusions

The key conclusion of this study is the importance of the coverage ratio of dot actuators. Not only is the coverage ratio a critical parameter for the achievable strain, but it is also of utmost importance when modeling the electro-active behavior of such actuators. The achievable strain in circular DEAs always decreases with raising coverage ratios, resulting in a maximum of displacement close to 50% radial coverage. 

## Figures and Tables

**Table 1:** Comparison of radii and resulting active-to-passive ratios in previous studies.

Study	Electrode Radius (mm)	Total DE Radius (mm)	Coverage Ratio
Wissler and Mazza [15]	7.5	75	0.1
Lee et al. [14]	15	48	0.3125
Yin et al. [13]	25	120	0.2083
Sahu et al. [12]	25	100	0.25

**Table 2:** Parameters of the hyperelastic Yeoh material model.

Yeoh Parameter	Fit Value (Pa)
$C_{1}$	178,426
$C_{2}$	−6023
$C_{3}$	4234

## Data Availability

Data are contained within the article.

## Abbreviations

The following abbreviations are used in this manuscript:

DEA	Dielctric elastomer actuator
DE	Dielectric elastomer
